# The prognostic role of maximum tumor dissemination derived by PET/CT in oncological diseases: a systematic review

**DOI:** 10.3389/fmed.2025.1726567

**Published:** 2026-01-05

**Authors:** Slavko Tasevski, Giorgio Treglia, Andreea Marin, Francesco Bertagna, Domenico Albano

**Affiliations:** 1 University Institute for Positron Emission Tomography, Skopje, North Macedonia; 2 Division of Nuclear Medicine, Imaging Institute of Southern Switzerland, Ente Ospedaliero Cantonale, Bellinzona/Lugano, Switzerland; 3 Faculty of Biology and Medicine, University of Lausanne, Lausanne, Switzerland; 4 Faculty of Biomedical Sciences, Università della Svizzera italiana, Lugano, Switzerland; 5 Clinic of Nuclear Medicine Central University Emergency Military Hospital “Dr Carol Davila”, Bucharest, Romania; 6 Nuclear Medicine, University of Brescia, Brescia, Italy; 7 Nuclear Medicine Department, ASST Spedali Civili di Brescia, Brescia, Italy

**Keywords:** Dmax, FDG, lymphoma, nuclear medicine, PET, PET/CT

## Abstract

**Background/Objectives:**

Maximum tumor dissemination (Dmax) measured by positron-emission tomography/computed tomography (PET/CT) is a semiquantitative parameter recently introduced with potential prognostic role in several oncological diseases. It is defined as a three-dimensional feature that represents the maximal distance between the two farthest hypermetabolic PET lesions. The aim of our systematic review is to investigate the effective role of Dmax in the management of oncological patients.

**Methods:**

The current systematic review was carried out following a preset protocol, and the “Preferred Reporting Items for a Systematic Review and Meta-Analysis” served as a guideline for its development. A comprehensive search of the PubMed/MEDLINE, Embase and Cochrane library databases was conducted until August 2025.

**Results:**

A total of 37 studies were included in our research. Lymphoma was the most frequent cancer investigated, followed by prostate cancer, lung cancer and breast cancer. Despite their heterogeneity, most studies showed a significant prognostic role of Dmax in predicting overall survival (OS) and progression free survival (PFS). The combination of Dmax with other PET features, especially MTV, seemed to be useful to stratify patients risk of relapse and/or death.

**Conclusions:**

Despite several limitations affecting this analysis, especially related to the heterogeneity of the studies included, PET/CT seems to have a prognostic impact in several oncological diseases, especially in lymphoma. However, few methodological issues still need to be solved before we can implement Dmax in clinical practice.

## Introduction

2-deoxy-2-[18F]-fluoro-D-glucose (2-[^18^F]FDG) positron emission tomography/computed tomography (PET/CT) plays a crucial role in the management of several cancers in different settings, such as staging disease, planning radiotherapy, assessing response to therapy, predicting prognosis (1). To move past purely visual/qualitative PET evaluation, numerous semiquantitative PET parameters have been developed to capture diverse facets of the disease, including surrogate of uptake (standardized uptake value, SUV), patient body composition (e.g., sarcopenic index) and the total tumor burden (e.g., metabolic tumor volume and texture features) (2, 3). SUV is a measurement used in PET scans to quantify the concentration of a radioactive tracer in a specific tissue, normalized by the injected dose and the patient's body features [such as body weight, body surface area (BSA) or lean body mass]. It was the most easy and frequent variable applied but with many limitations (4). Particularly, SUV measurement is directly affected many factors, such as patients features (weight, body composition), scanner characteristics, kind of protocols used, risk of extravasation, size. Instead, MTV is defined as the total volume of all metabolic active lesions and has been extensively studied in lymphoma, with its prognostic value repeatedly demonstrated but with several methodological issues (5, 6). The choice of threshold method (SUV as absolute value, SUV rate) to derive MTV is crucial and not universally shared. Despite the introduction of specific software for its measurement, the procedure is time consuming and operator-dependent.

Though these varied parameters offer encouraging prognostic insights for progression-free and overall survival (OS), their clinical utility is currently limited. The field awaits the standardization of their measurement and the establishment of shared methodology. Only through confirmatory prospective validation studies in defined patient groups these promising PET-based biomarkers could be successfully integrated into clinical practice.

Recently, another 2-[^18^F]FDG PET/CT–based prognostic factor that has gained increasing attention is the maximum tumor dissemination (Dmax), defined as the greatest distance between two metabolically active lesions. Most studies to date confirm its association with patient survival (7). However, there is lack of standardization of the methodology for its measurement. While MTV provides an estimate of the total metabolic burden, it does not inherently capture the spatial distribution or dissemination pattern of the disease. A patient with several clustered lesions may have the same MTV as a patient with widely disseminated, solitary lesions, yet their clinical outcomes and potential treatment strategies may differ significantly. Therefore, a metric that quantifies the maximum distance between lesions, such as Dmax, could offer a unique and intuitive reflection of disease spread, potentially correlating with more aggressive tumor biology and poorer outcomes.

The aim of this systematic review is to investigate the prognostic role of Dmax across different cancer types and using different PET radiopharmaceuticals.

## Methods

### Protocol

The current systematic review was carried out following a preset protocol, and the “Preferred Reporting Items for a Systematic Review and Meta-Analysis” (PRISMA 2020 statement) served as a guideline for its development and reporting (8). The PRISMA checklist is available in Supplementary Table S1.

As a first step, a direct review query using the Population, Intervention, Comparator, and Outcomes (PICO) framework was done: “What is the prognostic role (‘outcome') of Dmax measured by PET/CT using different radiopharmaceuticals (‘intervention') in patients with oncological diseases (‘population') compared or not to other PET features (‘comparator')?” Two investigators (D.A. and S.T.) independently performed the literature search, the study selection, the data extraction and the quality evaluation. In case of disagreements, a third opinion (G.T.) was asked.

### Search strategy

A comprehensive literature search of the PubMed/MEDLINE, Scopus, and Embase databases was conducted to find relevant published articles about the role of PET/CT in patients affected by oncological diseases. Furthermore, subsequent research on the ClinicalTrials.gov database for ongoing investigations (access date: 1 August 2025) was done.

We used a search algorithm based on a combination of the following terms: (1) “PET” OR “positron emission tomography” AND (2) “Dmax” OR “tumor dissemination” or “tumor distance.”

No beginning date limit was used for our literature search, which was updated until August 1, 2025. To enlarge our research, all the references of the retrieved articles were also screened searching for additional articles.

### Study selection process

Studies or subsets in studies investigating the value of Dmax measured on PET/CT in patients with different oncological diseases were eligible for inclusion. Exclusion criteria were: (1) articles not in the field of interest; (2) review articles, meta-analyses, letters, conference proceedings, and editorials; and (3) case reports or small case series (less than 10 patients included), to minimize the risk of bias from under-powered studies. Two researchers (S.T. and D.A.) independently reviewed the titles and abstracts of the articles, applying the above-mentioned inclusion and exclusion criteria, and the same two readers then independently reviewed the full-text version of the research to evaluate their suitability.

### Data extraction process and collection

For every included study, data were collected concerning the basic study features (first author name, year of publication, country, funding source, and study design), technical variables (PET device used, metabolic features analyzed, and software used), the main clinical patient characteristics (number of patients, age, gender, and type of cancer), and the main findings. The main data of the articles included in this review were represented in Tables and in the “Results” section.

Meta-analysis (quantitative synthesis) was not performed as significant heterogeneity among the selected studies (such as the different samples, cancers or PET radiopharmaceuticals) was expected. Progression-free survival (PFS) and overall survival (OS) were defined according to data provided by the authors of the original articles as the time interval from the initial diagnosis until disease relapse, progression, death, or the last follow-up for PFS, and as a time interval from the initial diagnosis until death or the last follow-up for OS.

### Quality assessment (risk of bias assessment)

A quality assessment of included articles was performed to analyze the risk of bias in individual studies to the review query. Four domains (patient selection, index test, reference standard, and flow and timing) were evaluated for risk of bias. At the same time, three sectors were assessed for applicability concerns (patient selection, index test, and reference standard) by using the QUADAS-2 tool (9).

## Results

### Literature search and study selection

**Figure 1:**
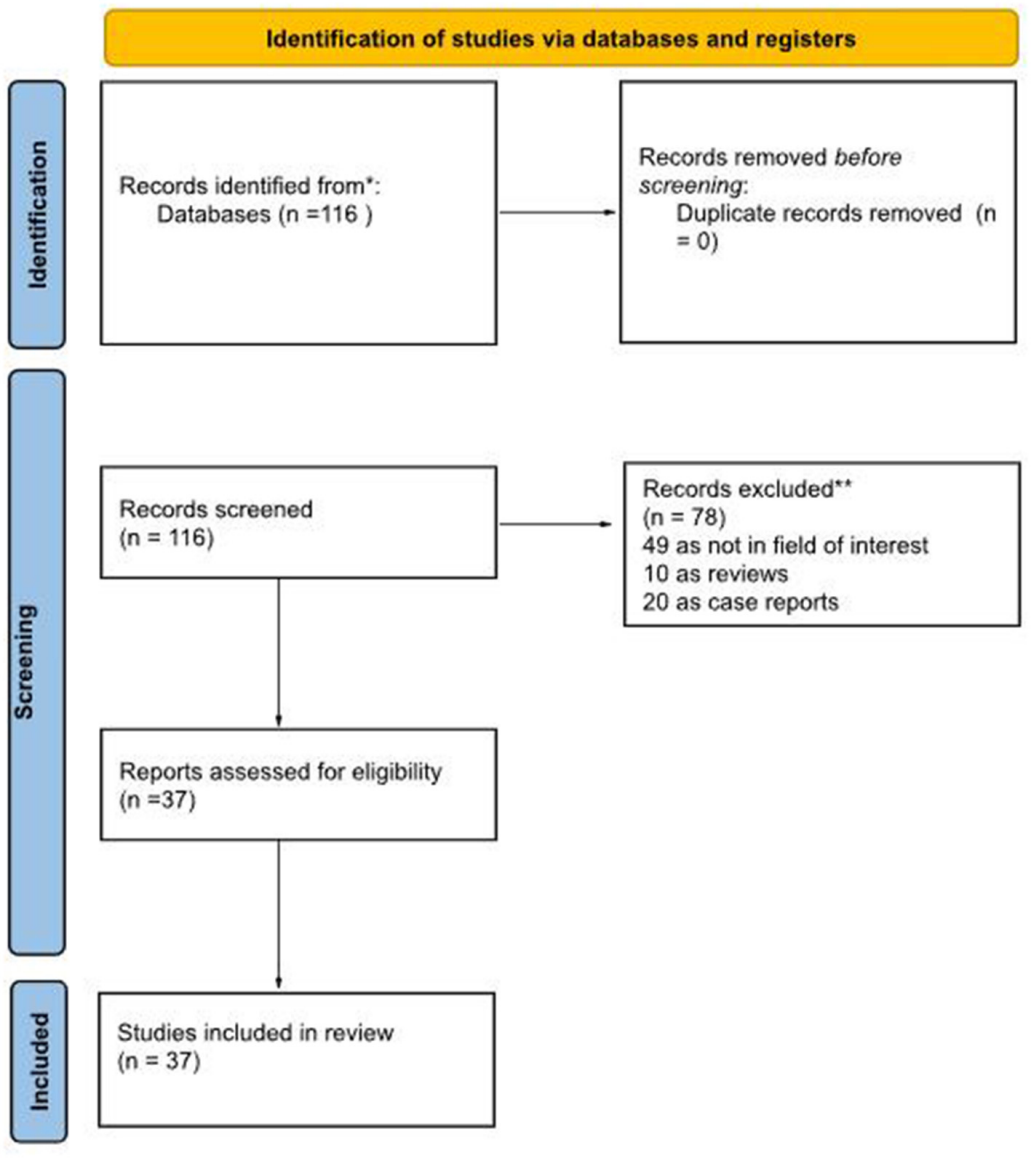
Literature search flowchart.

Our literature search, last updated on 1 August 2025, initially yielded 116 records. After applying our inclusion and exclusion criteria, we excluded 79 records. The reasons for exclusion were: 49 records were outside the field of interest; 10 records were identified as reviews, letters or editorials; 20 were case reports. Ultimately, 37 records were eligible for a full-text assessment and were included in the systematic review (qualitative synthesis) (10–46). A further check of the references within these selected articles did not reveal any additional manuscripts for inclusion. Figure 1 summarizes the study selection process.

### Studies and patients characteristics

The main features of the 37 included studies in the systematic review are summarized in Tables 1–3 (10–46). Regarding general study information (Table 1), all articles were published after 2020 in Europe and Asia. All studies but two had a retrospective design, and thirteen of these articles declared funding in their text.

**Table 1:** Studies' general information.

**First author**	**Year**	**Country**	**Funding source**	**Study design**	**Kind of cancer**	**No. of patients**
Cottereau AS (10)	2020	France	None declared	R	Lymphoma (DLBCL)	95
Zhou Y (11)	2021	China	None declared	R	Lymphoma (HL)	65
Cottereau AS (12)	2021	France	None declared	R	Lymphoma (DLBCL)	290
Cottereau AS bis (13)	2021	France	None declared	R	Lymphoma (DLBCL)	290
Durmo R (14)	2022	Italy	GRADE Onlus; Associazione Italiana per la Ricerca sul Cancro; Italian Ministry of Health Ricerca Corrente Annual Program 2023	R	Lymphoma (HL)	155
Li H (15)	2022	China	National Natural Science Foundation of China (No. 81771866)	R	Lymphoma (FL)	126
Aksu A (16)	2022	Turkey	None declared	R	Prostate cancer (adenocarcinoma - mCRPC)	38
Aksu A bis (17)	2022	Turkey	None declared	R	Prostate cancer (adenocarcinoma)	41
Eertink JJ (18)	2022	The Netherland	Dutch Cancer Society (# VU 2018–11648)	P	Lymphoma (DLBCL)	317
Eertink JJ bis (19)	2022	The Netherland	Dutch Cancer Society (# VU 2018–11648)	P	Lymphoma (DLBCL)	296
Girum KB (20)	2022	France	None declared	R	Lymphoma (DLBCL)	382
Gong H (21)	2022	China	None declared	R	Lymphoma (AITL)	81
Vergote KJV (22)	2023	Belgium	None declared	R	Lymphoma (MCL)	83
Aksu A (23)	2023	Turkey	None declared	R	Lymphoma (HL)	52
Dang J (24)	2023	China	Science & Technology Department of Sichuan Province (No. 22ZDYF1359), Sichuan Medical Health and Health Care Promotion Institute (KY2022SJ0260) and Sichuan Cancer Hospital Outstanding Youth Funding (YB 2023022)	R	Lymphoma (DLBCL)	154
Ferrandez MC (25)	2023	The Netherland	Hanarth Fonds Fund and the Dutch Cancer Society (#VU-2018–11648)	R	Lymphoma (DLBCL)	296 (HOVON-84) + 340 (PETAL)
Jo JH (26)	2023	Korea	None declared	R	Lymphoma (DLBCL)	63
Liu C (27)	2023	China	National Natural Science Foundation of China (No. 82102173) and the 2021 Shandong Medical Association Clinical Research Fund: Qilu Special Project (No. YXH2022X02198)	R	Lymphoma (DLBCL)	139
Peng X (28)	2023	China	Science and Technology Program of Sichuan Province (Grant No.22DYF2372); Science and Technology Program of Sichuan Province (Grant No.2020YFS0417); Sichuan Medical Research Project (Grant No. S21030); Sichuan Cancer Hospital Outstanding Youth Funding (Grant No. YB2021029); and Wu Jieping Medical Foundation Clinical Research Special Fund Project (Grant No.320.6750.19094-36)	R	Lymphoma (DLBCL)	181
Rodier C (29)	2023	France	None declared	R	Lymphoma (FL)	201
Tan W (30)	2023	China	Shandong Provincial Natural Science Foundation (Grant NO. ZR2021LZL005, ZR2019LZL019), the National Natural Science Foundation of China (Grant NO. 82172866), the grants from the Department of Science & Technology of Shandong Province (Grant NO. 2021CXGC011102), and the Start-up fund of Shandong Cancer Hospital (2020PYA04)	R	Lung cancer (NSCLC)	101
Wang F (31)	2023	China	Foundation of Changzhou Sci&Tech Program (Grant No. CJ20200118, CJ20210075), Changzhou High-Level Medical Talents Training Project (NO:2016ZCLJ024), and Key project of Jiangsu Province Health Committee (ZD2021043)	R	Lymphoma (DLBCL)	253
Xie Y (32)	2023	China	None declared	R	Lymphoma (PTCL)	95
Xu H (33)	2023	China	None declared	R	Lymphoma (DLBCL)	113
Albano D (34)	2024	Italy	None declared	R	Lymphoma (BL)	78
Lasnon C (35)	2024	France	None declared	R	Breast cancer	66
Mouheb M (36)	2024	France	None declared	R	Lymphoma (HL)	166
Yang T (37)	2024	China	Huai'an Science and Technology Project (grant no. HAB202017 to WT), The Innovation Key Talent Project of the Hospital (Grant No. ZC202208 to Weijing Tao)	R	Lymphoma (DLBCL)	424
Albano D (38)	2025	Italy	None declared	R	Prostate cancer	164
Albano D bis (39)	2025	Italy	None declared	R	Lymphoma (MCL)	120
Cui Y (40)	2025	China	National Natural Science Foundation (Nos. 81471695, 81971655, 82027804, 82001873). Shanxi Province Higher Education “Billion Project” Science and Technology Guidance Project (IDD/SXMU-2024-02), Four “Batches” Innovation Project of invigorating Medical through Science and Technology of Shanxi Province (No. 2022XM38), Central leading local science and Technology Development Fund Project (No. YDZJSX2022A058) and supported by Fundamental Research Program of Shanxi Province (No.202303021221226)	R	Lymphoma (DLBCL)	86
Aksu A (41)	2025	Turkey	None declared	R	Lymphoma (DLBCL)	90
Dondolin R (42)	2025	Italy	None declared	nr	Lymphoma (DLBCL)	120
Jiang Q (43)	2025	China	National Natural Science Foundation of China (Nos. U22A20290 and 82170180 to B.X.; No. 82470187 to J.Z.; and No. 82100204), the Natural Science Foundation of Fujian Province, China (Nos. 2023J06054 and 2021J011359 to J.Z.), and the Xiamen Municipal Bureau of Science and Technology (No. 3502Z20224011 to B.X.; and No. 3502Z20234001 to J.Z.)	R	Lymphoma (FL)	155
Pellegrino S (44)	2025	Italy	European Union—Next Generation EU—NRRP M6C2—Investment 2.1 Enhancement and strengthening of biomedical research in the NHS—PNRR-MCNT2-2023-12377713, CUP C63C24000370006 and only partly by the Associazione Italiana per la Ricerca sul Cancro (AIRC), Grant Number IG 2021—ID, 25945 project—	R	Lung cancer (NSCLC)	78
Seban RD (45)	2025	France	None declared	R	Breast cancer	128
Mirshahvalad SA (46)	2025	Canada	None declared	R	Lymphoma (DLBCL)	51

R, retrospective; P, prospective; HL, Hodgkin lymphoma; DLBCL, diffuse large B-cell lymphoma; FL, follicular lymphoma; AITL, angioimmunoblastic T-cell lymphoma; PTCL, peripheral T-cell lymphoma; MCL, mantle cell lymphoma; BL, Burkitt lymphoma; nr, not reported.

**Table 2:** Patients' general information.

**First author**	**Average/median age (range)**	**M:F**	**Early:advanced stage**	**Main findings**
Cottereau AS (10)	46 (18–59)	53:42	0:95	Dmax significantly correlated with PFS and OS. The combination of MTV and Dmax helped to stratify patients
Zhou Y (11)	29 (8–72)	45:20	36:29	Dmax significantly correlated with PFS and OS
Cottereau AS (12)	(60–80)	170:120	26:264	SDmax significantly correlated with PFS and OS. The combination of MTV and Dmax helped to stratify patients
Cottereau AS bis (13)	(60–80)	170:120	26:264	SDmax significantly correlated with PFS and OS, despite the methods
Durmo R (14)	nr	79:76	77:78	Dmax significantly correlated with PFS. The combination of interim PET response and Dmax helped to stratify patients
Li H (15)	53 (21–76)	63:63	22:104	Dmax significantly correlated with PFS
Aksu A (16)	67	38	0:38	Lower Dmax in the progressed group. Dmax was the only prognostic factor of treatment response in comparison with other PET parameters
Aksu A bis (17)	69 (53–85)	41	nr	Strong correlation between Dmax and PSA, PSMA-TVtotal, TL-PSMAtotal
Eertink JJ (18)	65 (23–80)	161:156	51:266	Dmaxbulk was one of the best predictors of treatment outcome
Eertink JJ bis (19)	65 (55–72)	152:144	48:248	Dmax and realted features were significantly correlated with PFS
Girum KB (20)	62.1 (34–73)	207:175	nr	Dmax significantly correlated with PFS and OS. The combination of MTV and Dmax helped to stratify patients
Gong H (21)	63	53:28	5:76	Dmax significantly correlated with PFS and OS. The combination of MTV and Dmax helped to stratify patients
Vergote KJV (22)	66 (58–72)	62:21	12:71	Dmax not significantly correlated with PFS and OS
Aksu A (23)	39	31:21	19:33	No significant correlation in Dmax and Dmaxvox with interim PET response. No significant difference in Dmax and Dmaxvox between progressive and non-progressive group
Dang J (24)	56 (16–87)	78:76	56:98	Dmax is an independent risk factor for PFS. The combination of %ΔSUVmax and Dmax helped to stratify patients
Ferrandez MC (25)	nr	nr	nr	A weak association for Dmaxbulk with P(TTP1) was found for HOVON-84. Moderate association for Dmaxbulk with P(TTP1) was found for PETAL. Higher P(TTP1) is related with higher Dmaxbulk values
Jo JH (26)	57.3 (21–87)	28:35	24:39	Dmax significantly correlated with time to progression
Liu C (27)	nr	78:61	41:98	SDmax significantly correlated with PFS
Peng X (28)	nr	90:91	70:105	Dmax significantly correlated with PFS. The combination of Dmax with gender, Ann Arbor stage, pathology type, number of extranodal involvement, LDH level and MTV, helped to stratify patients
Rodier C (29)	64 (30–88)	144:57	82:119	No significant correlation with TLT
Tan W (30)	60 (50–67)	59:42	0:101	Dmax significantly correlated with PFS and OS. The combination of Dmax and MTV can improve survival prediction
Wang F (31)	65 (13–91)	130:123	87:166 Lugano 95:158 Ann Arbor	Dmax significantly correlated with PFS and OS. Combination of Dmax and ECOG >/=2 helped to stratify patients
Xie Y (32)	64 (16–84)	59:46	10:85	Dmax significantly correlated with PFS and OS
Xu H (33)	61 (28–83)	57:56	26:87	Dmax and SDmax significantly correlated with PFS. SDmax was an independent predictor prognostic factor of PFS
Albano D (34)	52.8 (18–80)	51:27	22:56	Dmax significantly correlated with OS, not with PFS
Lasnon C (35)	60 (32–93)	nr	0:66	Dmax significantly correlated with PFS
Mouheb M (36)	nr	88:78	101:65	Dmax is not significantly correlated with PFS
Yang T (37)	nr	194:147	115:226	Dmax significantly correlated with PFS and OS. Combination of Dmax with tMTV and radiomic features helped to stratify patients
Albano D (38)	70.4 (48–87)	164:0	56:108	Dmax significantly correlated with PFS
Albano D bis (39)	65.6 (30–89)	90:30	5:115	Dmax significantly correlated with OS
Cui Y (40)	57.8	45:41	43:43	ΔDmax% significantly correlated with PFS. Combination of ΔDmax% and ΔtMTV% helped to stratify patients
Aksu A (41)	61 (23–88)	62:28	34:56	There is a significant difference of Dmax values between progressive and non-progressive patients. Combination of Dmax and other PET parameters with or without clinical parameters integrated in machine learning models helped to stratify patients
Dondolin R (42)	67 (51–76)	50:70	33:87	Dmax significantly correlated with PFS, OS, with Ann Arbor stage and IPI. Combination of Dmax with or without other PET parameters and high ctDNA levels helped to stratify patients
Jiang Q (43)	nr	74:81	43:112	Dmax significantly correlated with PFS and POD24. Combination of Dmax with tMTV and LDH helped to stratify patients
Pellegrino S (44)	64 (38–84)	55:23	0:78	Dmax significantly correlated with PFS and OS. Combination of Dmax with tMTV helped to stratify patients
Seban RD (45)	57 (23–85) T-DXd cohort	nr	0:128	Low Dmax significantly correlated with overall response rate in T-Dxd cohort. Dmax significantly correlated with PFS in both cohorts. Dmax significantly correlated with OS only in SG cohort. Combination of Dmax with tTMTV helped to stratify patients for PFS and OS in the SG cohort and PFS in the T-DXd cohort
Mirshahvalad SA (46)	56.1	32:19	8:43	Dmax significantly correlated with PFS

M, male; F, female; HL, Hodgkin lymphoma; DLBCL, diffuse large B-cell lymphoma; FL, follicular lymphoma; AITL, angioimmunoblastic T-cell lymphoma; PTCL, peripheral T-cell lymphoma; MCL, mantle cell lymphoma; Nr, not reported; PFS, progression-free survival; OS, overall survival; TTP, time to progression; MTV, metabolic tumor volume; TLG, total lesion glycolysis; TLT, time to lymphoma treatmente; SDmax, Dmax normalized by body surface area. POD24, progression of disease within 24 months; LDH, lactate dehydrogenase.

**Table 3:** Main technical features of the scanner and protocols.

**First author**	**Radiotracer**	**Type of PET scanner**	**Radiotracer activity injected, (MBq)**	**Uptake time (min)**	**Software**	**Dmax cutoff (cm)**	**Other PET features**
Cottereau AS (10)	[18F]FDG	nr	nr	nr	LIFEx	45	SUVmax, MTV, TLG, spread features
Zhou Y (11)	[18F]FDG	Discovery STE; (GE Medical Systems, Milwaukee WI, USA)	4.07–5.55/kg	60 ± 10	LIFEx	57.4	SUVmin, SUVmax, SUVmean, SUVpeak, SUVst, MTV, TLG, Dmax, histogram-derived features, shape-derived features, and texture features
Cottereau AS (12)	[18F]FDG	nr	nr	nr	LIFEx	47	MTV
Cottereau AS bis (13)	[18F]FDG	nr	nr	71.7 ± 14.1	LIFEx	nr	MTV
Durmo R (14)	[18F]FDG	nr	nr	nr	LIFEx & Fiji	20^***^	MTV, TLG
Li H (15)	[18F]FDG	Discovery VCT system (GE Healthcare, Milwaukee, WI, USA)	3.7–4.4/kg	60	R	56.73	SUVmax, MTV, TLG
Aksu A (16)	[68Ga]PSMA	Gemini TF (Philips, Eindhoven, The Netherlands)	115	60	LIFEx	79.8	SUVmax, PSMA-TV, TL-PSMA
Aksu A bis (17)	[68Ga]PSMA	Ingenuity TF 64 (Philips Medical Systems, Cleveland OH, USA)	185	45 ± 5 min Delayed imaging 45 min after the first imaging in the pelvic region	LIFEx	nr	PSMA-TVtotal, TL-PSMAtotal, prostate SUVmax, PSMA-TVprostate, texture features
Eertink JJ (18)	[18F]FDG	nr	nr	nr	RaCat	nr	SUVmax, SUVmean, SUVpeak, MTV, TLG, SPREAD and texture features
Eertink JJ bis (19)	[18F]FDG	nr	nr	nr	RaCat	nr	SUVmax, SUVmean, SUVpeak, MTV, TLG, SPREAD and texture features
Girum KB (20)	[18F]FDG	nr	nr	nr	LIFEx	59	MTV
Gong H (21)	[18F]FDG	Biograph 16-slice High Resolution (Siemens, Germany)	3.7–5.55/kg	60	LIFEx	65.7	MTV
Vergote KJV (22)	[18F]FDG	Biograph 16 HiRez, Siemens Truepoint 40 (Siemens Healthcare, Erlangen, Germany) and Discovery MI4 (GE Healthcare, Chicago, IL)	3–4.25/kg	60	MIM	60^***^	SUVmax, SUVmean, SUVpeak, MTV, TLG,
Aksu A (23)	[18F]FDG	Discovery 710, (GE Medical Systems, Wisconsin, USA)	3.7/kg	60	LIFEx	nr	MTV/DmaxVox, TLG/DmaxVox, SUVmax, MTV, TLG
Dang J (24)	[18F]FDG	Biograph MCT-64 ((Siemens Healthcare, Erlangen, Germany))	4.0/kg	60	LIFEx	53.2	SUVmax, tMTV, tTLG, %ΔSUVmax, %ΔtMTV, %ΔtTLG, StMTV, StTLG, Deauville score
Ferrandez MC (25)	[18F]FDG	nr	nr	nr	ACCURATE	nr	MTV
Jo JH (26)	[18F]FDG	GEMINI and GEMINI TF 64 (Philips Medical Systems, Cleveland, OH, USA)	nr	nr	LIFEx	27.5	SUVmax, SUVmean, tMTV, TLG
Liu C (27)	[18F]FDG	nr	nr	nr	AccuContour version 3.2;ManteiaTech	13.5	tMTV
Peng X (28)	[18F]FDG	Biograph mCT (Siemens Healthcare, Erlangen, Germany)	3.7–5.55/kg	60	LIFEx	53.9	SUVmax, MTV, TLG
Rodier C (29)	[18F]FDG	nr	nr	nr	AW Server, General Electrics, Milwaukee, USA	32	tMTV
Tan W (30)	[18F]FDG	Discovery LS (GE Healthcare, Milwaukee, WI, USA)	370	60	LIFEx	48.5	SUVmax, SUVmean, TLG, MTV
Wang F (31)	[18F]FDG	Biograph mCT, (Siemens Healthcare, Erlangen, Germany)	4.44/kg	45–60	Nr for Dmax. For MBV, (Syngo TrueD System Siemens Healthcare)	45.34	MBV
Xie Y (32)	[18F]FDG	Gemini GXL	5.18/kg	nr	LIFEx	65.95	SUVmax, MTV, TLG
Xu H (33)	[18F]FDG	Discovery VCT-64 (GE Healthcare, Milwaukee, USA)	3.7–5.5/kg	40–60	AW 4.7 workstation, LIFEx	57.8	MTV
Albano D (34)	[18F]FDG	Discovery ST and a Discovery 690 (GE)	3.5–4.5/kg	60	LIFEx	33.4	SUVbw, SUVlbm, SUVbsa, MTV and TLG
Lasnon C (35)	[18F]FDG	TrueV Biograph (Siemens Healthineers USA) and VEREOS (Philips Medical Solutions, USA)	3/kg	nr	Syngo.via and LIFEx	18.1	SUVpeak, TLG, MTV, PERCIST
Mouheb M (36)	[18F]FDG	Discovery ST (GE Healthcare), Biograph mCT (Siemens Healthineers), Biograph mCT flow (Siemens Healthineers) and Discovery MI (GE Healthcare)	nr	60	Syngo.via	15.9	SUVmax, tMTV, TLG, Dbulk
Yang T (37)	[18F]FDG	Biograph 16 (Siemens Healthcare, Erlangen, Germany) and Gemini GXL (Philips Corp, Netherlands)	3.70–5.55/kg	60 ± 5	LIFEx	22	tMTV, Radscore
Albano D (38)	[18F]PSMA	Discovery ST and a Discovery 690 (GE)	305	90	LIFEx	15.66^*^	PSMA-TV, PSMA-TTV, PSMA-TL, PSMA-TTL
Albano D bis (39)	[18F]FDG	Discovery ST and a Discovery 690 (GE)	3.5–4.5/kg	60	LIFEx	48	SUVbw, SUVlbm, SUVbsa, MTV and TLG
Cui Y (40)	[18F]FDG	Discovery MI (GE Healthcare)	3.7–5.55/kg	50–70	LIFEx	96.47%^**^	ΔSUVmax%, ΔMTV%, ΔTLG%, Deauville score
Aksu A (41)	[18F]FDG	Discovery 710 (GE Medical Systems, Waukesha, Wisconsin, USA)	3.7/kg	60 ± 10	LIFEx	28.3	SUVmax, tMTV, tTLG, MBV
Dondolin R (42)	[18F]FDG	nr	2.5–3/kg	60 ± 10	LIFEx	39	tMTV, tTLG, SUVmax
Jiang Q (43)	[18F]FDG	Discovery Molecular Imaging (MI) system (GE Healthcare, Milwaukee/Waukesha, WI, USA), Gemini GXL, UM780 and discovery clarity 710.	5.18/kg	30	LIFEx	64.24	tMTV, tTLG, SUVmax
Pellegrino S (44)	[18F]FDG	Ingenuity TF (Philips Healthcare, Best, the Netherlands)	370	60	LIFEx	34.4 for PFS 8.8 for OS	tMTV, tTLG, SUVmax, SUVmean, MTV, TLG
Seban RD (45)	[18F]FDG	Vereos (Philips) and Biograph Vision 600 (Siemens)	196	54–78	LIFEx	34^***^ from SG cohort 54.4^***^ from T-DXd cohort	tMTV, SUVmax
Mirshahvalad SA (46)	[18F]FDG	Biograph mCT 40 (Siemens Healthineers, Erlangen, Germany)	5/kg	60	Mirada XD Workstation, Mirada Medical	14	SUVmax, SUVmean, SUVpeak, tMTV, tTLG, SUVmax-to-liver ratio, SUVmean-to-liver ratio, Deauville score

SUV, standardized uptake value; MTV, metabolic tumor volume; TLG, total lesion glycolysis; SA, surface area.

^*^SDmax, Dmax normalized by body surface area; PFS, progression-free survival; OS, overall survival; T-DXd, Trastuzumab Deruxtecan; SG, Sacituzumab Govitecan.

^**^ΔDmax%.

^***^Median values of Dmax.

The performance of Dmax derived from PET/CT was investigated in different oncological conditions with lymphoma as most frequent cancer (*n* = 30), followed by prostate cancer (*n* = 3), lung cancer (*n* = 2) and breast cancer (*n* = 2). Among lymphoma, the most representative histotype was DLBCL (*n* = 18), followed by HL (*n* = 4) and FL (*n* = 3).

Participant ages ranged from a median/mean of 29–70.4 years, usually showing a female predominance. There was a prevalence of advanced stage disease compared with early stage disease.

In almost all studies, [^18^F]FDG was the radiotracer used. Only for articles including prostate cancer, [^68^Ga]PSMA and [^18^F]PSMA were the radiopharmaceuticals.

Methodologically, the average injected radiotracer activity varied considerably. When expressed as relative value, the administered activity ranged from 3 to 5.5 MBq/kg; as absolute activities, it ranged from 115 to 370 MBq. Consistently across all investigations, the time between injection and scan was approximately 60 min.

Different software was used for the measurement of Dmax, but LIFEx was the most common (47). The methodology for measuring Dmax varied, though the LIFEx software platform was most commonly employed (47). The process typically involved semi-automated segmentation of hypermetabolic lesions, often using a fixed SUV threshold (e.g., SUVmax ≥4.0 or 41% of SUVmax) or an adaptive method, followed by automatic calculation of the maximum distance between the centroids of the two farthest lesions in three-dimensional space. This highlights a potential source of methodological variation, as different segmentation methods can influence the final Dmax value.

In addition to Dmax, other semi-quantitative PET parameters were calculated, including SUVmax, MTV, total lesion glycolysis (TLG), and other texture features.

In some cases, Dmax was normalized by body surface area (BSA) and was called SDmax, changing the unit of measurement (12, 13, 22, 27, 29, 34). Also for this reason, the thresholds derived from Dmax (or SDmax) were very heterogeneous among studies. With these limitations, Dmax ranged from 14 to 79.8 cm. Among semiquantitative parameters, SUVmax was the most commonly measured PET feature, followed by MTV and total lesion glycolysis (TLG).

### Risk of bias and applicability

**Figure 2:**
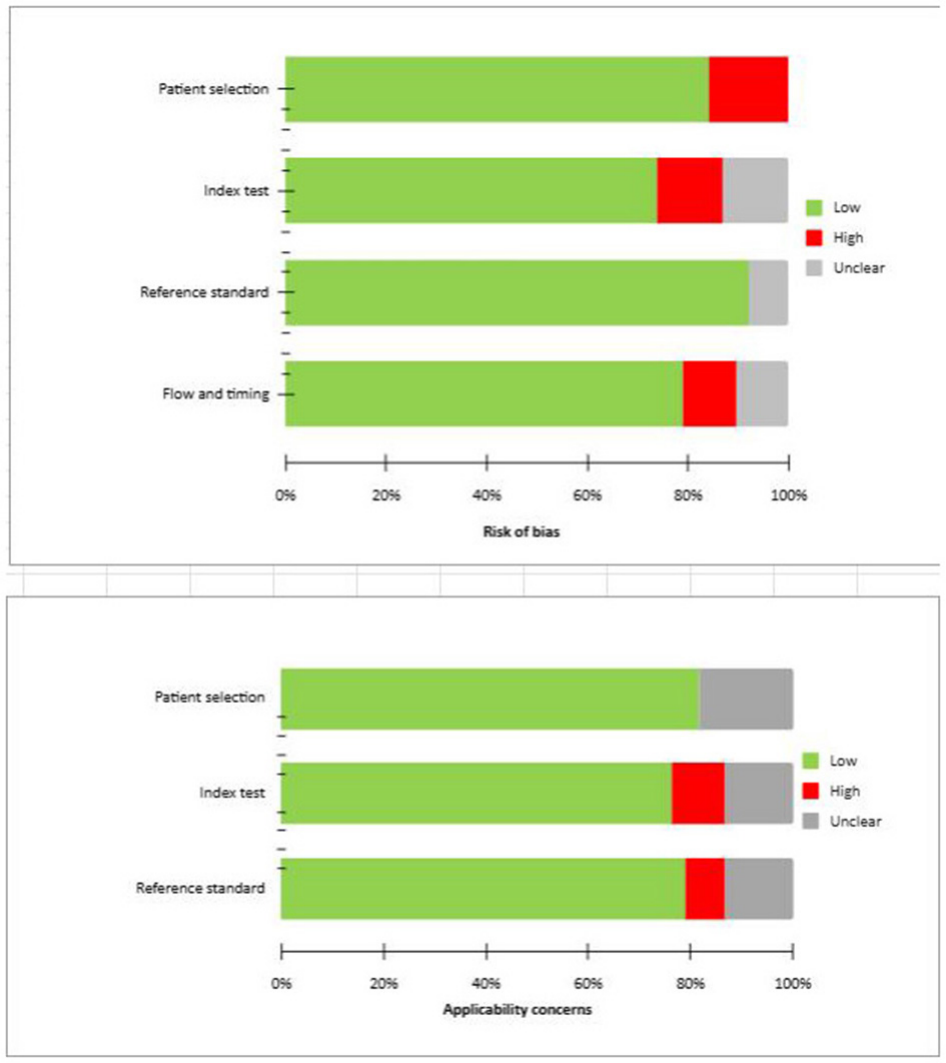
QUADAS 2 scores of the articles included.

The overall assessment of the risk of bias and concerns about the applicability of the included papers according to QUADAS-2 are provided in Figure 2.

### Primary results of the included studies

Regarding the prognostic role of 2-[^18^F]FDG PET/CT, in almost all studies Dmax showed to be an independent prognostic factor for PFS (10–15, 19–21, 24, 26–28, 30–32, 35, 37, 38, 40–46) and OS (10–13, 19–21, 30–32, 34, 37, 39, 42, 44, 45). In all studies, Dmax or SDmax were analyzed as an absolute value, except of one study (40) where ΔDmax% was evaluated. On the other hand, in only three studies (23, 34, 36) Dmax demonstrated no significant role in predicting PFS. These studies recruited BL (34) and HL (23, 36) patients.

Concerning prostate cancer, in both studies (16, 38) investigating prognostic role of Dmax the findings are positive. Among studies including breast cancer (35, 45) and lung cancer (30, 44), Dmax confirmed to be an independent prognostic factor in all cases.

In combination with Dmax, the most frequent metabolic variable with a prognostic role was MTV (10, 12, 20, 21, 28, 30, 37, 43–45).

## Discussion

The advent of [^18^F]FDG PET/CT has revolutionized the management of various cancers, moving beyond mere anatomical assessment to provide crucial metabolic insights for staging, therapy response, and prognostication (1). While established semi-quantitative parameters like SUV, MTV, and TLG have demonstrated value, their widespread clinical translation has been hampered by significant methodological biases, including inter-operator, inter-scanner, and reconstruction parameter variability (47). In this context, our systematic review aimed to critically evaluate the emerging prognostic role of Dmax, a novel PET-derived parameter, across different oncological diseases (2, 3). Dmax emerges as a simpler, more robust alternative variable. Dmax is a simple three-dimensional PET feature that represents the distance between the centroids of two lesions, inherently reflecting the spatial dissemination of the disease. Dmax may partially avoid the technical and operator-dependent biases that affect other PET metrics, including scanner and reconstruction parameters, but it remains directly related to the segmentation of disease. Moreover, several softwares that can measure automatically Dmax with good accuracy and reproducibility are currently available. However, until now, no studies investigated the reproducibility in Dmax measurements. Dmax seems to offer an intuitive metric for tumor heterogeneity and potentially superior staging power compared to the stage system.

Our comprehensive search identified 37 studies, predominantly focusing on lymphoma (*n* = 30), followed by prostate (*n* = 3), lung (*n* = 2), and breast cancer (*n* = 2). The first simple evidence is the fact that Dmax was investigated almost exclusively in lymphoma (30/37 studies) and the radiotracer most investigated was FDG (34/37 studies). A striking finding was the consistent demonstration of Dmax as a significant independent prognostic factor for both OS and PFS across the majority of these studies, despite their inherent heterogeneity. This robust association, particularly prominent in lymphoma, underscores Dmax's potential as a valuable, non-invasive biomarker. The ability of Dmax to intuitively represent the patient-based spatial migration and spread of the disease offers a unique dimension beyond traditional measures of metabolic activity or tumor volume. Furthermore, the combination of Dmax with other PET features, especially MTV, appeared to enhance patient risk stratification for relapse and/or death, suggesting a synergistic effect when multiple parameters characterizing different aspects of tumor biology are considered (10, 12, 20, 21, 28, 30, 37, 43–45). The prognostic strength of Dmax suggests it may add a valuable functional and quantitative dimension to traditional anatomical staging. For instance in lymphomas, within a single Ann Arbor stage (e.g., Stage IV), Dmax could potentially subdivide patients into groups with different outcomes based on the actual spread of their disease, thus refining risk stratification beyond what is possible with staging alone. The strong association between a larger Dmax and poorer survival likely reflects underlying tumor biology. A widely disseminated disease pattern may indicate a higher degree of diffusion between lymphatic system (typical of aggressive lymphomas) or vascular invasion (solid tumors), successful metastatic seeding, and immune evasion, all potentially hallmarks of aggressive malignancies. The “spread” of lymphoma may be a sign of systemic dissemination and infiltration throughout the body's existing lymphoid and reticuloendothelial tissues. Therefore, Dmax can be viewed not just as a geometric measure, but as an indirect biomarker of a tumor's invasive and metastatic potential.

One of the limitations of Dmax is in the investigation of early-stage disease, especially stage I, where other PET features (like MTV and TLG) remain viable. Furthermore, Dmax requires standardization, particularly regarding normalization for body size, and clear cut-off values before it can be routinely integrated into clinical practice for prognostic prediction (12, 13, 22, 27, 29, 34).

However, our systematic review also highlights several critical methodological issues that currently impede the definitive integration of Dmax into routine clinical practice. Firstly, the significant heterogeneity among the included studies is a major limitation. This heterogeneity spans various aspects, including differences in cancer types and histologies (even within lymphoma, various subtypes were studied), patient characteristics (e.g., advanced vs. early stage), radiotracer activity administration (ranging from 3 to 5.5 MBq/kg relative or 115 to 370 MBq absolute), and the specific software used for Dmax calculation. Such variability makes direct comparisons between studies challenging and precludes a quantitative meta-analysis, limiting the generalizability of the findings and hindering the establishment of universal Dmax thresholds. For example, Dmax (or SDmax) thresholds varied considerably (20–79.8 cm) across studies, underscoring the need for standardization.

Only in three studies (23, 34, 36) Dmax failed to be a prognostic factor and MTV showed to be superior than Dmax demonstrating to be significantly associated with survival.

The primary drawback of this systematic review was the considerable clinical and methodological heterogeneity found among the included studies. Due to this lack of uniformity, the authors opted not to perform a quantitative synthesis (meta-analysis). Nevertheless, a strict methodology was employed to ensure both transparency and reproducibility. The main findings of this evidence-based article are anticipated to be valuable for informing and suggesting future well-designed studies focusing on Dmax in patients with cancer.

## Conclusions

In conclusion, Dmax measured by PET/CT shows considerable promise as a non-invasive prognostic biomarker in various oncological diseases, especially lymphoma. Its intuitive representation of disease spread and its potential for higher reproducibility compared to other complex radiomic features are significant advantages. Nevertheless, before Dmax can be widely implemented in clinical practice, future research must address the existing methodological heterogeneity. This includes developing standardized acquisition and reconstruction protocols, establishing consistent Dmax calculation methodologies (including its normalization), defining universally accepted cut-off values, and conducting well-designed, prospective, multi-center validation studies across specific cancer types. Overcoming these challenges will pave the way for Dmax to become a valuable tool in personalized oncology, enhancing patient risk stratification and guiding therapeutic decisions.

Future research must prioritize the development of standardized operating procedures for Dmax calculation. This includes consensus on segmentation methods, recommendations for normalization (e.g., SDmax for body size), and the execution of well-designed, prospective, multi-center validation studies across specific cancer types. Only after addressing these challenges can Dmax be robustly integrated into clinical trials and eventually, routine practice.

## Data Availability

The original contributions presented in the study are included in the article/Supplementary material, further inquiries can be directed to the corresponding author.

## References

[1] Boellaard R Herrmann K Barrington SF Cranston I Mottaghy FM Hoekstra CJ . [18F]FDG PET/CT: EANM procedure guidelines for tumour imaging: version 3.0. EANM J. (2025) 1:100006. doi: 10.1016/j.eanmj.2025.100006

[2] Albano D Ravanelli M Durmo R Versari A Filice A Rizzo A . Semiquantitative 2-[18F]FDG PET/CT-based parameters role in lymphoma. Front Med. (2024) 11:1515040. doi: 10.3389/fmed.2024.1515040 39744526 PMC11688351

[3] Albano D Dondi F Ravanelli M Tucci A Farina D Giubbini R . Prognostic role of “radiological” sarcopenia in lymphoma: a systematic review. Clin Lymphoma Myeloma Leuk. (2022) 22:e340–9. doi: 10.1016/j.clml.2021.11.006 34893457

[4] Lodge MA. Repeatability of SUV in oncologic 18F-FDG PET. J Nucl Med. (2017) 58:523–32. doi: 10.2967/jnumed.116.186353 28232605 PMC5373499

[5] Agarwal A Wehrle CJ Satish S Mahajan P Kamath S Koyfman S . PET-Assessed metabolic tumor volume across the spectrum of solid-organ malignancies: a review of the literature. Biomedicines. (2025) 13:123. doi: 10.3390/biomedicines13010123 39857707 PMC11762135

[6] Boellaard R Buvat I Nioche C Ceriani L Cottereau AS Guerra L . International benchmark for total metabolic tumor volume measurement in baseline 18F-FDG PET/CT of lymphoma patients: a milestone toward clinical implementation. J. Nucl. Med. (2024) 65:1343–8. doi: 10.2967/jnumed.124.267789 39089812 PMC11372260

[7] Albano D Treglia G Dondi F Calabrò A Rizzo A Annunziata S . 18F-FDG PET/CT maximum tumor dissemination (Dmax) in lymphoma: a new prognostic factor? Cancers. (2023) 15:2494. doi: 10.3390/cancers15092494 37173962 PMC10177347

[8] Page MJ McKenzie JE Bossuyt PM Boutron I Hoffmann TC Mulrow CD . The PRISMA 2020 statement: an updated guideline for reporting systematic reviews. BMJ. (2021) 372:n71. doi: 10.1136/bmj.n71 33782057 PMC8005924

[9] Whiting PF Rutjes AW Westwood ME Mallett S Deeks JJ Reitsma JB . QUADAS-2 Group. QUADAS-2: a revised tool for the quality assessment of diagnostic accuracy studies. Ann Intern Med. (2011) 155:529–36. doi: 10.7326/0003-4819-155-8-201110180-00009 22007046

[10] Cottereau AS Nioche C Dirand AS Clerc J Morschhauser F Casasnovas O . 18F-FDG PET dissemination features in diffuse large B-cell lymphoma are predictive of outcome. J. Nucl. Med. (2020) 61:40–5. doi: 10.2967/jnumed.119.229450 31201248 PMC6954460

[11] Zhou Y Zhu Y Chen Z Li J Sang S Deng S. Radiomic features of 18F-FDG PET in hodgkin lymphoma are predictive of outcomes. Contrast Media Mol Imaging. (2021) 2021:6347404. doi: 10.1155/2021/6347404 34887712 PMC8629643

[12] Cottereau AS Meignan M Nioche C Capobianco N Clerc J Chartier L . Risk stratification in diffuse large B-cell lymphoma using lesion dissemination and metabolic tumor burden calculated from baseline PET/CT. Ann. Oncol. (2021) 32:404–11. doi: 10.1016/j.annonc.2020.11.019 33278600

[13] Cottereau AS Meignan M Nioche C Clerc J Chartier L Vercellino L . New approaches in characterization of lesions dissemination in DLBCL patients on baseline PET/CT. Cancers. (2021) 13:3998. doi: 10.3390/cancers13163998 34439152 PMC8392801

[14] Durmo R Donati B Rebaud L Cottereau AS Ruffini A Nizzoli ME . Prognostic value of lesion dissemination in doxorubicin, bleomycin, vinblastine, and dacarbazine-treated, interimPET-negative classical Hodgkin Lymphoma patients: a radio-genomic study. Hematol Oncol. (2022) 40:645–57. doi: 10.1002/hon.3025 35606338 PMC9796042

[15] Li H Wang M Zhang Y Hu F Wang K Wang C . Prediction of prognosis and pathologic grade in follicular lymphoma using 18F-FDG PET/CT. Front Oncol. (2022) 12:943151. doi: 10.3389/fonc.2022.943151 35965552 PMC9366037

[16] Aksu A Vural Topuz Ö Yilmaz B Karahan Sen NP Acar E Çapa Kaya G. Prediction of early biochemical response after 177Lu-PSMA radioligand therapy with 68Ga-PSMA PET, a different perspective with quantitative parameters. Nucl Med Commun. (2022) 43:468–74. doi: 10.1097/MNM.0000000000001539 35045552

[17] Aksu A Vural Topuz Ö Yilmaz G Çapa Kaya G Yilmaz B. Dual time point imaging of staging PSMA PET/CT quantification; spread and radiomic analyses. Ann Nucl Med. (2022) 36:310–8. doi: 10.1007/s12149-021-01705-5 34988888

[18] Eertink JJ van de Brug T Wiegers SE Zwezerijnen GJC Pfaehler EAG Lugtenburg PJ . 18F-FDG PET baseline radiomics features improve the prediction of treatment outcome in diffuse large B-cell lymphoma. Eur J Nucl Med Mol Imaging. (2022) 49:932–42. doi: 10.1007/s00259-021-05480-3 34405277 PMC8803694

[19] Eertink JJ Zwezerijnen GJC Cysouw MCF Wiegers SE Pfaehler EAG Lugtenburg PJ . Comparing lesion and feature selections to predict progression in newly diagnosed DLBCL patients with FDG PET/CT radiomics features. Eur J Nucl Med Mol Imaging. (2022) 49:4642–51. doi: 10.1007/s00259-022-05916-4 35925442 PMC9606052

[20] Girum KB Rebaud L Cottereau AS Meignan M Clerc J Vercellino L . 18F-FDG PET maximum-intensity projections and artificial intelligence: a win-win combination to easily measure prognostic biomarkers in DLBCL patients. J. Nucl. Med. (2022) 63:1925–32. doi: 10.2967/jnumed.121.263501 35710733 PMC9730929

[21] Gong H Tang B Li T Li J Tang L Ding C. The added prognostic values of baseline PET dissemination parameter in patients with angioimmunoblastic T-cell lymphoma. EJHaem. (2023) 4:67–77. doi: 10.1002/jha2.610 36819177 PMC9928789

[22] Vergote VKJ Verhoef G Janssens A. Woei-A-Jin FJSH, Laenen A, Tousseyn T, et al. [18F]FDG-PET/CT volumetric parameters can predict outcome in untreated mantle cell lymphoma. Leuk Lymphoma. (2023) 64:161–70. doi: 10.1080/10428194.2022.2131415 36223113

[23] Aksu A Küçüker KA Solmaz S Turgut B. A different perspective on PET/CT before treatment in patients with Hodgkin lymphoma: importance of volumetric and dissemination parameters. Ann Hematol. (2024) 103:813–22. doi: 10.1007/s00277-023-05547-1 37964021

[24] Dang J Peng X Wu P Yao Y Tan X Ye Z . Predictive value of Dmax and %ΔSUVmax of 18F-FDG PET/CT for the prognosis of patients with diffuse large B-cell lymphoma. BMC Med Imaging. (2023) 23:173. doi: 10.1186/s12880-023-01138-8 37907837 PMC10617085

[25] Ferrández MC Golla SSV Eertink JJ de Vries BM Lugtenburg PJ Wiegers SE . An artificial intelligence method using FDG PET to predict treatment outcome in diffuse large B cell lymphoma patients. Sci Rep. (2023) 13:13111. doi: 10.1038/s41598-023-40218-1 37573446 PMC10423266

[26] Jo JH Chung HW Kim SY Lee MH So Y FDG. PET/CT maximum tumor dissemination to predict recurrence in patients with diffuse large B-Cell lymphoma. Nucl Med Mol Imaging. (2023) 57:26–33. doi: 10.1007/s13139-022-00782-2 36643943 PMC9832207

[27] Liu C Shi P Li Z Li B Li Z. A nomogram for predicting the rapid progression of diffuse large B-cell lymphoma established by combining baseline PET/CT total metabolic tumor volume, lesion diffusion, and TP53 mutations. Cancer Med. (2023) 12:16734–43. doi: 10.1002/cam4.6295 37366281 PMC10501242

[28] Peng X Yu S Kou Y Dang J Wu P Yao Y . Prediction nomogram based on 18F-FDG PET/CT and clinical parameters for patients with diffuse large B-cell lymphoma. Ann Hematol. (2023) 102:3115–24. doi: 10.1007/s00277-023-05336-w 37400729

[29] Rodier C Kanagaratnam L Morland D Herbin A Durand A Chauchet A . Risk factors of progression in low-tumor burden follicular lymphoma initially managed by watch and wait in the era of PET and rituximab. Hemasphere. (2023) 7:e861. doi: 10.1097/HS9.0000000000000861 37125257 PMC10146112

[30] Tan W Zhang Y Wang J Zheng Z Xing L Sun X . PET/CT tumor dissemination characteristic predicts the outcome of first-line systemic therapy in non-small cell lung cancer. Acad Radiol. (2023) 30:2904–12. doi: 10.1016/j.acra.2023.03.027 37202226

[31] Wang F Cui S Lu L Shao X Yan F Liu Y . Dissemination feature based on PET/CT is a risk factor for diffuse large B cell lymphoma patients outcome. BMC Cancer. (2023) 23:1165. doi: 10.1186/s12885-023-11333-z 38030989 PMC10687880

[32] Xie Y Teng Y Jiang C Ding C Zhou Z. Prognostic value of 18F-FDG lesion dissemination features in patients with peripheral T-cell lymphoma (PTCL). Jpn J Radiol. (2023) 41:777–86. doi: 10.1007/s11604-023-01398-y 36752954

[33] Xu H Ma J Yang G Xiao S Li W Sun Y . Prognostic value of metabolic tumor volume and lesion dissemination from baseline PET/CT in patients with diffuse large B-cell lymphoma: further risk stratification of the group with low-risk and high-risk NCCN-IPI. Eur J Radiol. (2023) 163:110798. doi: 10.1016/j.ejrad.2023.110798 37030099

[34] Albano D Calabrò A Talin A Dondi F Pagani C Tucci A . [18]F FDG PET/CT dissemination features in adult burkitt lymphoma are predictive of outcome. Ann Hematol. (2024) 103:2419–27. doi: 10.1007/s00277-024-05672-5 38374254

[35] Lasnon C Morel A Aide N Da Silva A Emile G. Baseline and early 18F-FDG PET/CT evaluations as predictors of progression-free survival in metastatic breast cancer patients treated with targeted anti-CDK therapy. Cancer Imaging. (2024) 24:90. doi: 10.1186/s40644-024-00727-2 38982546 PMC11232230

[36] Mouheb M Pierre-Jean M Devillers A Fermé C Benchalal M Manson G . Prognostic value of baseline tumor burden and tumor dissemination extracted from 18 F-FDG PET/CT in a cohort of adult patients with early or advanced Hodgkin lymphoma. Clin Nucl Med. (2024) 49:e1–5. doi: 10.1097/RLU.0000000000004930 38015041

[37] Yang T Sun Z Shi Y Teng Y Cheng L Zhu R . Development and validation of prognostic models based on 18F-FDG PET radiomics, metabolic parameters, and clinical factors for elderly DLBCL patients. Ann Hematol. (2024) 103:5583–98. doi: 10.1007/s00277-024-06071-6 39480583

[38] Albano D Temponi A Bertagna F Suardi N Talin A Bonù ML . The prognostic role of staging [18F]PSMA-1007 PET/CT volumetric and dissemination features in prostate cancer. Ann Nucl Med. (2025) 39:518–26. doi: 10.1007/s12149-025-02026-7 39961974 PMC12014769

[39] Albano D Bianchetti N Talin A Dondi F Re A Tucci A . Prognostic role of pretreatment tumor burden and dissemination features from 2-[18F]FDG PET/CT in advanced mantle cell lymphoma. Hematol Oncol. (2025) 43:e70009. doi: 10.1002/hon.70009 39614626 PMC11607474

[40] Cui Y Li Y Hu W Wu Z Li S Wang H. Evaluating ΔMTV%, ΔDmax%, and %ΔSUVmax of 18F-FDG PET/CT for mid-treatment efficacy and prognosis in diffuse large B-cell lymphoma. Discover Oncology. (2025) 16:411. doi: 10.1007/s12672-025-02126-w 40146454 PMC11950622

[41] Aksu A Us A Küçüker KA Solmaz S Turgut B. Assessment of quantitative staging PET/computed tomography parameters using machine learning for early detection of progression in diffuse large B-cell lymphoma. Nucl Med Commun. (2025) 46:972–9. doi: 10.1097/MNM.0000000000002023 40583566

[42] Dondolin R Garrou F Almasri M Terzi Di Bergamo L Cosentino C Bruscaggin A . Integration of [18F]FDG-PET radiomics with liquid biopsy improves outcome prediction in newly diagnosed diffuse large B-cell lymphoma. Leukemia. (2025) 39:2207–14. doi: 10.1038/s41375-025-02688-2 40629066 PMC12380602

[43] Jiang Q Lin Z Chen Q Lin F Jiang C Deng M . Integration of PET/CT parameters and a clinical variable to predict the risk of progression of disease within 24 months (POD24) in follicular lymphoma. Quant Imaging Med Surg. (2025) 15:2468–80. doi: 10.21037/qims-24-1504 40160607 PMC11948388

[44] Pellegrino S Fonti R Morra R Di Donna E Servetto A Bianco R . Prognostic value of tumor dissemination (Dmax) derived from basal 18F-FDG positron emission tomography/computed tomography in patients with advanced non-small-cell lung cancer. Biomedicines. (2025) 13:477. doi: 10.3390/biomedicines13020477 40002890 PMC11853205

[45] Seban RD Champion L De Moura A Lerebours F Loirat D Pierga JY . Pre-treatment [18F]FDG PET/CT biomarkers for the prediction of antibody–drug conjugates efficacy in metastatic breast cancer. Eur J Nucl Med Mol Imaging. (2025) 52:708–18. doi: 10.1007/s00259-024-06929-x 39373900

[46] Mirshahvalad SA Kohan A Kulanthaivelu R Ortega C Metser U Hodgson D . Prognostic value of early post-treatment 18F-FDG PET/CT in diffuse large B-cell lymphoma patients receiving chimeric antigen receptor T-cell therapy. Cancer Imaging. (2025) 25:70. doi: 10.1186/s40644-025-00888-8 40484967 PMC12147336

[47] Nioche C Orlhac F Boughdad S Reuzé S Goya-Outi J Robert C . LIFEx: a freeware for radiomic feature calculation in multimodality imaging to accelerate advances in the characterization of tumor heterogeneity. Cancer Res. (2018) 78:4786–9. doi: 10.1158/0008-5472.CAN-18-0125 29959149

